# Heartburn as a Marker of the Success of Acid Suppression Therapy in Chronic Cough

**DOI:** 10.1007/s00408-021-00496-w

**Published:** 2021-11-19

**Authors:** H. Badri, I. Satia, V. Bansal, M. A. Mangi, A. Tangaroonsanti, K. R. DeVault, A. S. Lee, L. A. Houghton, J. A. Smith

**Affiliations:** 1 Division of Infection, Immunity and Respiratory Medicine, University of Manchester, Manchester, UK; 2 Department of Respiratory Medicine, North Manchester General Hospital, Manchester, UK; 3 McMaster University, Hamilton, ON Canada; 4 Division of Gastroenterology and Hepatology, Mayo Clinic, Jacksonville, FL USA; 5 Division of Pulmonary and Critical Care Medicine, Mayo Clinic, Rochester, MN USA; 6 Division of Cardiovascular Medicine, University of Toledo, Toledo, OH USA; 7 Liver and Digestive Institute, Samitivej Sukhumvit Hospital, Bangkok, Thailand; 8 Pulmonary Medicine, Mayo Clinic, Jacksonville, FL USA; 9 Division of Gastroenterology and Surgical Science, Leeds Institute of Medical Research at St James’s, University of Leeds, Leeds, UK; 10 Education and Research Centre 2nd floor, University of Manchester, Wythenshawe Hospital, Southmoor Rd, Manchester, M23 9LT UK

**Keywords:** Heartburn, Chronic cough, Gastro-oesophageal reflux disease, Acid suppression

## Abstract

**Purpose:**

Gastro-oesophageal reflux disease (GORD) is commonly thought to play an important role in chronic cough and patients are often empirically treated with acid suppression therapy. We sought to investigate the response rate to acid suppression treatment in patients with and without heartburn attending two specialist cough clinics.

**Methods:**

A retrospective review of 558 consecutive patients referred to two specialist cough clinics was performed (UK and USA). Patients who were treated with acid suppression were included and their documented response to treatment was collected. Binary logistic regression was used to ascertain the value of reported heartburn in predicting the response of chronic cough to acid suppression therapy.

**Results:**

Of 558 consecutive referrals, 238 patients were excluded due to missing data or cough duration of < 8 weeks. The remaining 320 patients were predominantly female (76%), with mean age 61 yrs (± 13) and 96.8% non-smokers, with chronic cough for 36 (18–117) months. Of 72 patients with heartburn, 20 (28%) noted improvement in their cough with acid suppression, whereas of 248 without heartburn, only 35 (14%) responded. Patients reporting heartburn were 2.7 (95% C.I. 1.3–5.6) times more likely to respond to acid suppression therapy (p = 0.007).

**Conclusion:**

In specialist cough clinics, few patients report a response of their chronic cough to acid suppression therapy. Nonetheless, heartburn is a useful predictor substantially increasing the likelihood of benefit.

## Introduction

Gastro-oesophageal reflux disease (GORD) is thought to play an important role in chronic cough [1–3]. Some studies estimate between 10% and 40% of chronic cough is related to GORD [4, 5]. Various mechanisms linking reflux and cough have been suggested, such as micro-aspiration of gastric content and/or stimulation of a vagal oesophago-bronchial reflex. [6, 7]. Validated measures demonstrating reflux reach the larynx/pharynx or biomarkers of micro-aspiration are lacking, making proximal reflux and micro-aspiration difficult to evaluate. However, several lines of evidence support the concept of reflux evoking cough via a vagal reflex, for example, Smith et al. 2010 have shown that in 48% of unselected chronic cough patients there were temporal associations between reflux and cough [5]. Interestingly, abnormalities of oesophageal motility associated with chronic cough demonstrate slow clearance of both swallowed food and refluxate. The longer residence of such material in the oesophagus may enhance stimulation of vagal reflexes [4].

The cost of treating atypical GORD symptoms, such as cough, with acid suppressants far outweighs the cost of treating typical GORD symptoms with studies quoting a 5.6 × greater cost [10]. Thus being able to select which chronic cough patients are more likely to respond to acid suppression is vital, in order to prevent excessive waste of resources and unnecessary risks associated with prolonged drug treatment for the patient. Treatment trials of acid suppressing therapy (proton pump inhibitors (PPIs), H2 antagonists ± alginates) used to be recommended as part of all guidelines for the management of patients with chronic cough [3]. Recent guidelines only recommend trials of acid-suppressant therapy in those with evidence of pathological reflux or heartburn. This is based on a retrospective analysis of pooled data from 9 randomised trials in patients thought to have extra-oesophageal symptoms of reflux, of which chronic cough was a subgroup [8]. This study suggested that therapeutic gain is most likely to be seen in those with abnormal 24-h pH monitoring or reported heartburn. These studies were all small (the largest was *n* = 39 patients) and therefore the total number of subjects in this analysis was only 163 and the predictive value of reflux symptoms and measures was not clear.

We set out to investigate the real-world response rate of acid suppression treatment in patients attending two specialist cough clinics and in particular which factors predicted likelihood of success of treatment.

## Materials and Methods

### Participants

Patients who had a cough persisting longer than 8 weeks, with documented evidence of treatment with antacid suppression therapy and no significant lung pathology were included. The response to treatment recorded in the notes was collected. As the degree of treatment response was not documented in a standard format, patients were categorised as responders if any improvement was documented. Any patients with missing duration of cough or response to treatment were excluded.

### Study Design

A retrospective review of the case notes of chronic cough patients attending two specialist cough services was conducted; one from the UK specialist tertiary clinic (Wythenshawe Hospital, Manchester University Foundation Trust, Manchester, UK) and the other from the US clinic (Mayo clinic, Jacksonville, Florida, USA) over a 12-month period from October 2013 to October 2014. Both clinics worked to very similar management protocols and based on the guidelines at the time, all patients received a trial of acid suppressing therapy.

Patients attending the Manchester clinic were referred from secondary care clinicians, whereas patients attending the US clinic could self-refer and were often referred directly from their primary care provider. As this was a review of clinical notes and the data were collected as part of routine care, no formal ethics approval was sought, but this review was registered with the local clinical audit and governance department. The Mayo clinic study required ethical approval and this was obtained (IRB 15-002903).

### Data Collection

Clinic proformas which captured data on age, sex, cough duration, lung function, treatment trials, and additional symptoms (such as heartburn) were used. Both groups of patients underwent history and examination by a physician. Routine investigations (radiology, full lung function tests, bronchial provocation tests, and bronchoscopy) were then carried out to exclude any significant causes of cough. Patients who had a persistent cough of greater than 8-week duration were included. The US clinic patients were seen by ear, nose, and throat specialists, pulmonology, and gastroenterologist, whereas the UK group were seen by pulmonology only. As part of their routine care, all patients then completed a treatment trial of dual acid suppression therapy (if not treated previously). This consisted of dual antacids in the form of PPIs (e.g. Omeprazole 20 mg BD/Lansoprazole 15 mg BD) plus a H2 antagonist (Ranitidine 300 mg OD) for a minimum of 8 weeks. The addition of nocturnal H2 antagonist to the dual PPI regimen is in order to enhance nocturnal gastric pH control and decrease nocturnal gastric acid breakthrough [9, 10] thus reducing the likelihood of reflux cough being triggered. Response of cough to acid suppression therapy was based on patient reported and physician judgement documented as yes/no response in the proforma.

### Statistical Analysis

The data were analysed using IBM SPSS version 22, 2013. Data were summarised as mean/median depending on the distribution. Mann–Whitney test was used to generate p value for non-normally distributed non-parametric data. Independent sample T test was used for the normally distributed data. Binary logistic regression was used to model the data with the dependent variable being recorded acid suppression response.

## Results

### Subjects

**Fig. 1:**
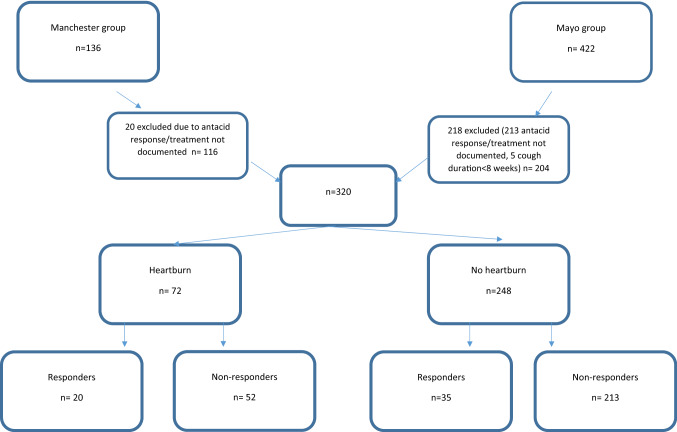
Summary of patient data analysed

Information was collected on 558, of those 238 patients were excluded (20 UK and 218 US). The reasons for exclusion were as follows: (i) the duration of cough not being documented, (ii) response to acid suppression not documented, and (iii) presence of heartburn not documented and patients not having chronic cough for > 8 weeks. Three hundred and twenty patients were included in final dataset (Fig. 1). Comparison of the two clinics indicated that there were some statistically significant differences between patients attending the UK clinic and the US clinic (Table 1). The US cohort was slightly older (64 ± 13 vs 57 ± 11.3 years), with a shorter duration of cough (30 vs 48 months) and reported less heartburn (16% vs 32.8%). The majority of patients were female (71%) and the mean age was 61 ± 13 years old. The median cough duration was 36 (12–120) months. Responders and non-responders of cough to acid suppression were well matched, with no significant differences in lung function (FEV1 and FVC), smoking status, sex, and age (Table 1).

**Table 1:** Patient characteristics across both clinics

	UK (*n* = 116)	US (*n* = 204)	*P*
Age (years)*	57 ± 11.3	64 ± 13.0	** <0.001**
Sex *Female (%)*	84 (72)	143(70)	0.756
Cough duration (months)**	48 (24–120)	30 (12–96)	** <0.001**
Smoking history			0.72
Current	2 (1.7)	6 (3)	
Ex-smokers	31 (26.7)	75 (37)	
Never smokers	81 (69.8)	123 (60)	
Missing data	2	0	
FEV1 percent predicted*	*n* = *76* 91.3 ± 22	*n* = *179* 86.3 ± 20.2	0.080
FVC percent predicted*	n = 69 100 ± 20.5	n = 179 92 ± 17.4	**0.002**
Heartburn	38 (32.8%)	34 (16%)	**0.002**
Acid suppression response	15 (12.9%)	40 (20%)	0.171

Statistically significant *P* values are given in bold

^*^Mean (SD)

^**^Median (IQR)

### Heartburn Symptoms and Acid Suppression Response

Only 72 (23%) patients reported heartburn and of these only 20 (28%) reported an improvement in cough. Of the 248 patients reporting no heartburn, 35 (14%) reported an improvement in cough in response to acid suppressing therapy. In the pooled cohort, only 17% (55/320) of all patients reported a response of their cough to acid suppression therapy (Fig. 1). There was no significant difference between patients from the two different clinics in terms of likelihood of success of acid suppression therapy. A chi-squared test showed that the presence of heartburn was significantly higher in responders than in non-responders (*p* = 0.007). The sensitivity of heartburn as a symptom in detecting response of cough to acid suppression was 36.4% and the specificity was 80.4%.

Binary logistic regression was carried out to ascertain the value of heartburn for predicting the likelihood of a response to acid suppression (Table 2). The model indicated that the presence of heartburn significantly predicted success of acid suppression therapy, *p* = 0.007. Furthermore, patients reporting heartburn were 2.7 times (95% C.I. 1.3–5.6) more likely to have a response of their cough to acid suppression therapy. A multivariate analysis of variables, such as age, sex, and lung function, did not have any significant effect on the likelihood of success of acid suppression, nor did the clinic site.

**Table 2:** Patient characteristics

	All patients *(n* = 320)	Responders *(n* = 55)	Non-responders *(n* = 265)	*p*
Age (years)*	61 ± 13	64 ± 14	61 ± 13	0.064
Female, *n (%)*	227(71)	35 (64)	192 (72)	0.126
FEV_1_ (percent predicted)*	88 (21)	86 (22)	88 (21)	0.494
FVC (percent predicted)*	94 (19)	92 (19)	95 (19)	0.343
Cough duration** (months)	36 (18–117)	36 (12–120)	36(18–96)	0.413
Heartburn *n (%)*	**72 (23)**	**20 (36%)**	**52 (20%)**	**0.007**
Smoking status				0.293
Never	204 (63.7)	38 (69.1)	166 (62.9)	
Ex	106 (33.1)	15 (27.3)	91 (34.3)	
Current	8 (2.5)	1 (1.8)	7 (2.6)	
Missing data	2	1	1	

Statistically significant *P* values are given in bold

Values are expressed as the mean ± SD for normally distributed continuous variables*, the median (IQR) for non-normally distributed data**, and frequency (percentage) for discrete variables. Chi-squared test was performed for parametric data. Significance: P ≤ 0.05. Mann –Whitney U test for non-parametric data. Significance: *P* ≤ 0.05

## Discussion

To our knowledge, this is the largest study of the effect of acid suppression on cough in patients with chronic cough. Our findings indicate that heartburn is the only significant predictor of success of acid suppression therapy when treating chronic cough in a ‘real-life’ setting. However, it must be acknowledged that only just over a third of those with heartburn gained benefit. [8, 10, 11].

Evidence that acid suppression improves cough in chronic cough patients is limited to studies with a small number of patients. Kahrilas et al. carried out a retrospective review and found nine RCTs on the effect of acid-suppressive therapy (either PPIs or the H2 antagonist ranitidine) on chronic cough symptoms [8]. Six of these studies did not report a significant improvement in cough, but two of these negative studies excluded patients with heartburn [11, 12]. It is also worth noting that the 3 of the negative studies included patients without pathological evidence of oesophageal acid exposure. Kahrilas et al. report that the therapeutic gain of treating chronic cough patients with acid-suppressive treatment was low and variable (between 8.6% and 35.8%) but is increased by selecting patients who report heartburn as a symptom. This is in keeping with the findings of our larger study. A more recent study of 312 patients with acute and sub-acute cough found that 46% had GORD symptoms, such as heartburn, which are significantly higher than in our study [13]. However, this may be due to the inclusion of a population with shorter duration of cough. The same group went on to investigate the effects of acid suppression treatment in patients with chronic cough in a smaller study (*n* = 37), selecting patients with both heartburn and cough and treating them with a PPI and prokinetic agent. Again, patients with GERD symptoms and cough improved significantly with acid suppression, consistent with our findings.

The most important finding of this study is the low response rate of chronic cough to acid suppression, even in the context of heartburn. Only 17% of the patients in this dataset reported a response of their cough to acid suppression which reflects the treatment refractory nature of chronic cough patients seen in specialist clinics [14]. Despite this low response rate, heartburn was a highly significant predictor of the likelihood of success of acid suppression therapy (*p* = 0.007) and resulted in a near threefold increase in the success of treatment. It is worth noting, however, that the vast proportion of patients with chronic cough with concomitant heartburn that responds to acid suppression are probably managed in the community and thus are far less likely to present to specialist clinics. A large epidemiological study looking at 14,669 individuals in a population-based cohort found that reported gastro-oesophageal reflux disease was a significant risk factor for chronic cough, especially in never and former smokers [17]. Another possible explanation for the low numbers of responders to acid suppression is that these therapies only suppress the acidity of the refluxate and have no effect on non-acid reflux or the number of reflux events. Decalmer et al. 2010 found that approximately 2/3 of reflux events in chronic cough patients were non-acid [5]. Other studies also report that chronic cough patients have an increased incidence of weak peristalsis with large breaks leading to poor oesophageal clearance of reflux [4, 5]. Thus, acid suppression alone may be ineffective at treating the commonest types of reflux in this patient group. In order to explore this further trials in chronic cough patients using therapies that target lower oesophageal sphincter relaxations, reducing non-acid and acid reflux and/or poor oesophageal clearance are needed.

Our dataset was typical of patients attending specialists cough clinics with the majority of patients being females between the ages of 50–60 years old, with normal spirometry and non-smokers. The patients in the responder vs non-responder group were well matched, with no significant differences between them (apart from reporting of heartburn). There were some statistically significant differences between the patients attending the US clinic and those attending the UK one (Table 2). The US group was older, had a lower FVC, and reported shorter duration of their cough than the UK group. However, none of these differences impacted our results. Although the US group was older they are still within the typical age group of chronic cough patients [14, 15]. The US clinic was based in Florida which is a popular retirement destination, and although we do not have data on where the patients attending the clinic came from, we speculate that the increased age may simply reflect the local population. The FVC percent predicted was slightly lower in the US patients; however, this did not reflect a clinically relevant difference. It is possible that there was a difference in body mass index between the patients attending the two clinics that can account for the difference in FVC; however, we do not have data on BMI for the UK patients. Subjects in the US group had a shorter duration of cough, but this is not clinically significant as these patients would still be classed as chronic cough patients. Furthermore, the duration of cough was not a significant factor between the responders and non-responders. One explanation for the difference in cough duration is that the US clinic accepts direct GP/primary care/self-referrals, whereas the UK clinic only accepts patients from secondary care. Thus, there is a delay from presentation to specialist clinic review (in the UK) which might account for the difference in cough duration. Twice as many patients in the UK group complained of heartburn compared to the US group (32.8% vs. 16%), although on the whole the numbers were small in both groups. The wider availability of over-the-counter acid suppression medication in the US and therefore the possibility of self-management of reflux compared to the UK could account this difference with patients successfully treating acid reflux-related chronic cough and avoiding the need for referral to specialist clinics. In general, UK patients need to be seen by a doctor prior to being prescribed acid suppression (at least for significant periods and at treatment doses), whereas in the US patients self-medication with these drugs is common, with treatment level doses being readily available over the counter. Despite the difference in the number of patients with heartburn in each group, this did not translate to a significant difference in the PPI response rate when the data were modelled.

At the time of collecting the data for this study, guidelines recommended treatment trials of acid suppression therapy as routine for all patients with chronic cough even in the absence of GORD symptoms, such as heartburn [16]. Our study data do not support this practice and suggested that acid suppression is unlikely to be successful in the majority of patients with chronic cough, which led to a change in practice in our centre. Since then, the guidelines have been updated and now discouraged routine empirical acid suppression use in these patients [17, 18].

Due to the observational and retrospective nature of this study the findings should be interpreted with some caution. As with all observational studies, misclassification of data or information bias when collecting the data may have occurred. Patients with incomplete or missing information on duration of cough, heartburn symptoms or duration of antacid therapy were excluded. The dosage of acid suppression therapy used was at the discretion of the prescribing clinician. However, both centres followed the current chronic cough guidelines [19, 20] on the management of GORD-related cough. Thus, although the dosing regimen may have differed between patients the treatment was in line with current guidelines and reflective of what happens in a real-life setting.

As this was a retrospective review, there were no validated endpoints. We used the reporting of heartburn and cough response as the main variables, which were both subjectively measured. There was no formal standardised way of measuring heartburn and thus this could vary between patients and clinicians and the degree of response was not recorded in a standard manner and therefore cannot be reported.

## Conclusion

This study suggests that heartburn is a predictor of the likelihood of chronic cough responding to acid suppression. Although this is consistent with previous retrospective analysis of clinical trial data, our analysis reveals that the overall proportion of patients with chronic cough responding acid suppression is low in a specialist cough setting. The majority of these patients’ cough was resistant to therapy.
